# Imagerie multimodale dans la rétinite ponctuée externe toxoplasmique: à propos d’un cas

**DOI:** 10.11604/pamj.2022.43.197.37953

**Published:** 2022-12-21

**Authors:** Malek Kharrat, Yoldez Ben Jemaa, Zeineb Kallel, Sana Sayadi, Walid Zbiba

**Affiliations:** 1 Département d’Ophtalmologie, Hôpital Mohamed Taher Maamouri Nabeul, Faculté de Médecine Université El Manar, Tunis, Tunisie

**Keywords:** Rétinite ponctuée externe, toxoplasmose oculaire, uvéite, imagerie multimodale, cas clinique, Punctuate outer retinitis, ocular toxoplasmosis, uveitis, multimodal imaging, case report

## Abstract

La toxoplasmose ponctuée de la rétine externe (RPE) est une variante rare de la choriorétinite toxoplasmique. Nous rapportons le cas d´une patiente de 21 ans se présentant pour un flou visuel de l´œil gauche (OG). L´examen trouvait une acuité visuelle corrigée (AVc) à 3/10^e^, un segment antérieur calme et un Tyndall vitréen à 1 croix. L´examen du fond de l´œil (FO) a montré un foyer blanc-jaunâtre supra-fovéolaire et de multiples lésions atropho-pigmentaires périphériques. L´AVc de l´œil adelphe était à 10/10^e^ avec un segment antérieur calme et au FO un foyer atropho-pigmentaire cicatriciel temporal supérieur. L´imagerie multimodale de l´OG a conclu à une RPE toxoplasmique. L´évolution sous traitement antibiotique et corticoïdes était favorable avec une AV finale à 10/10 à 10 jours. Ce cas illustre l´importance de l´imagerie multimodale dans la différenciation de la RPE toxoplasmique du syndrome des taches blanches et les autres causes de rétinites unilatérales.

## Abstract (en)

Punctuate Outer Retinal Toxoplasmosis (PORT) is a rare variant of toxoplasma chorioretinitis. We report the case of a 21-year-old patient presenting with visual blur of the left eye (LE). The examination found a corrected visual acuity (VA) at 3/10^th^, a quit anterior segment and a 1+ vitreous haze. Fundus examination showed a suprafoveolar yellowish-white lesions associated to multiple peripheral atrophic and pigmented ones. Visual acuity of the right eye was 10/10^th^ with a calm anterior segment. Fundus examination depicted an upper temporal cicatricial pigmented lesion. Multimodal imaging of LE objectified a PORT. The patient received antibiotic and corticosteroids with favorable clinical and functional outcome. Final VA reached 10/10 at day ten. This case illustrates the importance of multimodal imaging in the differentiation of PORT from the white dots syndrome and other unilateral retinitis.

## Introduction

La toxoplasmose oculaire est la principale cause d´uvéite postérieure chez l´homme touchant surtout le sujet jeune [1]. La forme clinique la plus fréquente est caractérisée par une uvéite postérieure récurrente unilatérale avec rétinite nécrosante. Les foyers de choroïdite toxoplasmique secondaires se développent à proximité des lésions cicatricielles choriorétinienne pigmentées et sont associées à une hyalite et une vascularite rétinienne. Plusieurs formes atypiques sont également décrites dans la littérature: les occlusions vasculaires, la neurorétinite, les taches de Roth, les granulomes du disque optique, le décollement sérieux rétiniens, les uvéites antérieures et intermédiaires et la rétinite ponctuée externe (RPE) [2]. Ces tableaux représentent un challenge diagnostique vu leur rareté d´où l´intérêt de l´imagerie multimodale en plus des investigations biologiques pour la mise en évidence de la maladie. Dans cet article nous présentons le cas d´une RPE toxoplasmique suspectée, diagnostiquée et prise en charge grâce aux données de l´imagerie multimodale.

## Patient et observation

**Informations relatives aux patients:** il s´agit d´une patiente âgée de 21 ans, sans d´antécédents pathologiques généraux ou ophtalmologiques notables, qui a consulté nos urgences pour une baisse brutale de la vision de l´œil gauche (OG) évoluant depuis une semaine.

**Figure 1:**
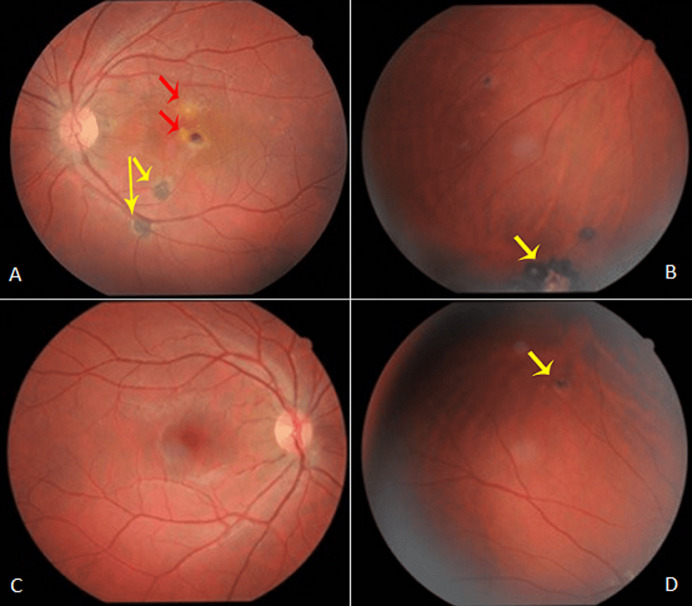
fond de l´œil (FO) des 2 yeux: A) foyers actifs (flèches rouges) et cicatriciels (flèches jaunes) du pôle postérieur et de l´extrême périphérie inférieure; B) de l´œil gauche; c-d: pôle postérieur; C) et foyer atropho-pigmentaire cicatriciel temporal supérieur, (D, flèche jaune) de l´œil droit

**Résultats cliniques:** l´examen de l´OG trouvait acuité visuelle corrigée (AVc) à 3/10^e^, un segment antérieur calme, une pression intraoculaire normale et un Tyndall cellulaire vitréen à 1+. L´examen du fond de l´œil (FO) montrait deux lésions punctiformes blanc-jaunâtre juxta-fovéolaire de 1/10^e^ et 1/5^e^ diamètre papillaire (Dp). D´autres foyers atropho-pigmentés étaient retrouvés en para papillaire, en périphérie rétinienne inférieure de part et d´autre l´arcade temporale (Figure 1A, Figure 1B). L´examen de l´œil droit (OD) trouvait une AVc à 10/10^e^, un segment antérieur calme avec au FO un foyer atropho-pigmentaire cicatriciel temporal supérieur de moins de 1/4 Dp situé à la moyenne périphérie (Figure 1C, Figure 1D).

**Figure 2:**
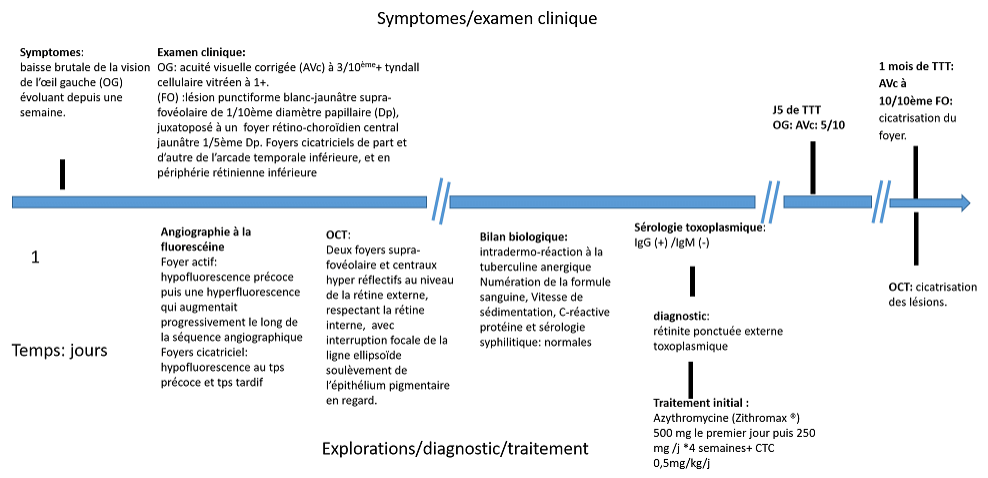
chronologie

**Chronologie:** la chronologie de la prise en charge de la patiente est présentée par la Figure 2.

**Figure 3:**
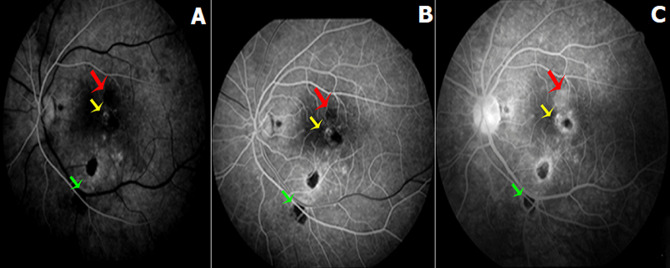
séquence angio graphique de l´OG: A) temps précoce; B) temps intermédiaire; C) temps tardif

**Figure 4:**
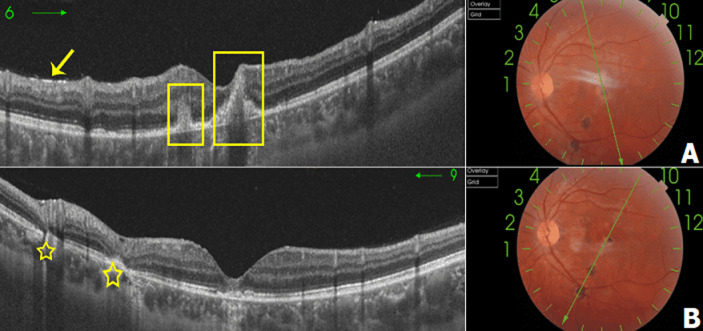
tomographie en cohérence optique (B scan) de l´OG passant par les différents foyers de l´OG: A) multiples petits points hyper réflectifs superficiels déposés sur la rétine interne (flèche jaune); B) les foyers inféro-maculaire se traduisaient par une atrophie et une désorganisation de la rétine externe avec des altérations du complexe EP-membrane de Bruch (étoiles)

**Figure 5:**
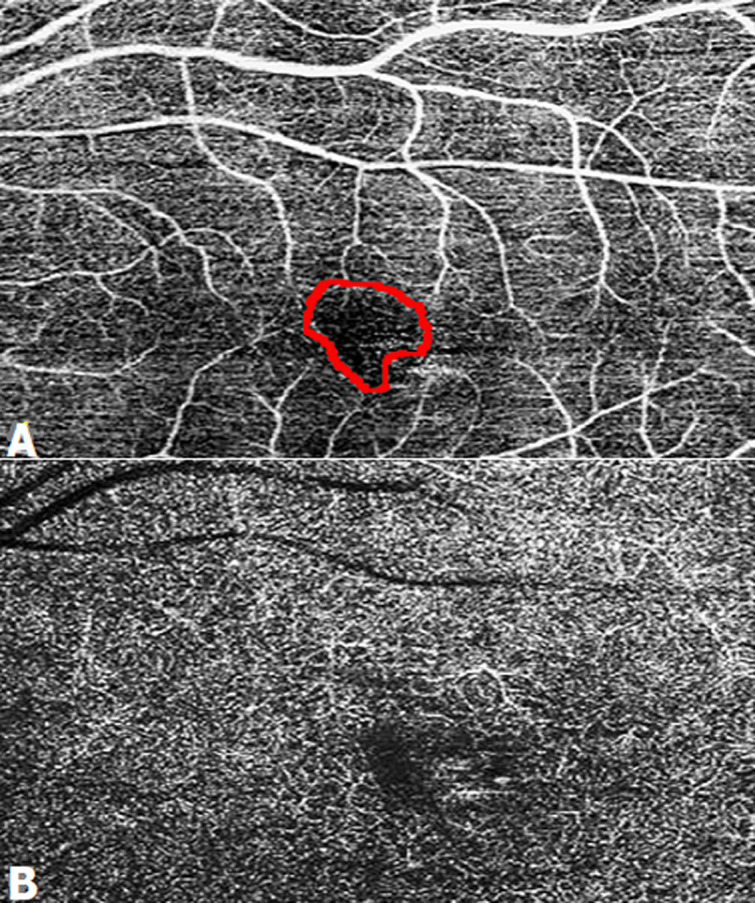
la tomographie par cohérence optique maculaire angiographie 6x6mm de l´OG: élargissement et irrégularité de la zone avasculaire centrale plus visible au niveau du plexus superficiel (A) que profond (B)

**Démarche diagnostique:** l´angiographie à la fluorescéine montrait une hypo fluorescence précoce des lésions centrales avec une hyper fluorescence progressive et diffusion tardive. Certaines lésions atropho pigmentaires de la périphérie rétinienne étaient hypo fluorescents en temps précoce puis hyper fluorescents au temps tardif correspondant à des foyers en cours de cicatrisation. Les autres foyers cicatriciels restaient hypo fluorescents le long de la séquence (flèche verte). Une hyper fluorescence papillaire était notée au temps tardif à l´OG (flèche jaune) (Figure 3). La tomographie par cohérence optique maculaire *Swept Source* (*SS-OCT*) objectivait de multiples petits points hyper réflectifs superficiels déposés sur la rétine interne (flèche jaune). Au niveau de la région fovéolaire, on notait la présence de deux foyers supra-fovéolaire et centraux hyper réflectifs au niveau de la rétine externe, respectant la rétine interne, avec interruption focale de la ligne ellipsoïde et soulèvement de l´épithélium pigmentaire en regard. Les foyers inféro-maculaire se traduisaient par une atrophie et une désorganisation de la rétine externe avec des altérations du complexe EP-membrane de Bruch associés à des microkystes optiquement vides, au niveau de la région fovéolaire, présence de deux foyers supra-fovéolaire et centraux hyper réflectifs au niveau de la rétine externe, respectant la rétine interne, avec interruption focale de la ligne ellipsoïde soulèvement de l´épithélium pigmentaire en regard (2 rectangles) (Figure 4). La tomographie par cohérence optique maculaire (*OCT*) angiographie révélait une interruption de la maille anastomotique périfovéolaire en temporal avec un élargissement de la zone avasculaire centrale plus marquée au niveau du plexus superficiel (Figure 5). Le bilan biologique trouvait une intradermo-réaction (IDR) à la tuberculine anergique, une numération de la formule sanguine ainsi qu´une vitesse de sédimentation, une CRP et une sérologie syphilitique normales. La sérologie toxoplasmique montrait un titre positif des IgG avec des IgM négatifs.

**Figure 6:**
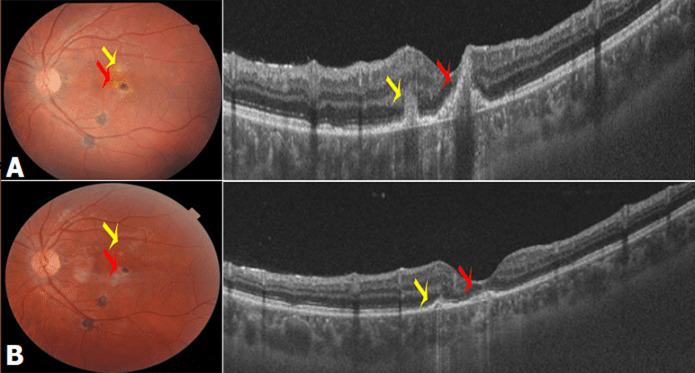
fond de l´œil et *OCT* du pôle postérieur de l´OG: A) avant traitement; B) 1 mois après traitement: diminution de la taille des foyers sur la photo FO marquée sur les coupes tomographiques en B scan (flèches jaunes)

**Intervention thérapeutique:** le diagnostic de rétinite ponctuée externe toxoplasmique (RPET) a été retenu, la patiente a été mise sous Azythromycine (Zithromax®) 500 mg le premier jour puis 250 mg par jour pendant 4 semaines. Nous avons associé une corticothérapie par voie orale à la dose de 0,5mg/kg/j au bout de 48 heures d´antibiothérapie.

**Suivi et résultats des interventions thérapeutiques:** l´AVC était remonté à 5/10 au bout du cinquième jour de traitement et à 10/10^e^ avec cicatrisation du foyer sur l´*OCT* maculaire à 1 mois de suivi.

**Point de vue de la patiente:** “À mon arrivée aux urgences, je croyais que je ne pourrai plus récupérer mon œil gauche. Grâce aux efforts de tout le staff médical, je peux lire de nouveau comme avant. J´espère que cet épisode ne se reproduira plus”.

**Consentement éclairé:** la patiente a donné son aval pour que nous puissions utiliser son bilan étiologique dans le rapport de ce cas.

## Discussion

Nous rapportons le cas d´une forme clinique rare de toxoplasmose oculaire diagnostiquée et prise en charge grâce à l´apport de l´imagerie multimodale. La toxoplasmose oculaire est la principale cause d´uvéite postérieure chez l´homme [1]. La forme typique est une rétinochoroïdite focale, touchant toute l´épaisseur rétinienne avoisinant souvent un foyer cicatriciel et associée à une réaction vitréenne assez importante [3]. La RPET est une variante rare mais assez importante de la toxoplasmose oculaire, se présentant sous la forme de lésions blanches grisâtres multifocales de la rétine externe et de l´épithélium pigmentaire surtout de la région maculaire [4]. Les formes congénitales sont généralement diagnostiquées pendant la 2^e^ ou la 3^e^ décade [3]. Dans notre cas, les nombreuses lésions cicatricielles au niveau du pôle postérieur et de l´extrême périphérie rétinienne, de différents stades évolutifs et la présence isolée des IgG spécifiques de la toxoplasmose, laissent penser à une forme congénitale, récurrente et asymptomatique de la maladie. Cette forme serait surtout l´apanage des immunocompétents. Elle est considérée comme un succès de la réponse immunitaire limitant l´extension de l´infection à la rétine interne [5].

L´imagerie multimodale était d´un apport considérable dans l´orientation diagnostique: l´angiographie à la fluorescéine a permis la détection de nombreux foyer sous fovéolaire peu visible au fond d´œil. Ces lésions rejoignent la description de Doft *et al*. [4] comme étant une hypo fluorescence précoce avec une hyper fluorescence tardive des lésions actives, peu intense du fait de leur localisation profonde au niveau du complexe rétine externe-EP avec une rétention papillaire possible Par ailleurs, de nombreuses publications ont rapporté l´aspect tomographique de la rétinite ponctuée externe toxoplasmique. Souza *et al*. [6] et Brandão-de-Resende *et al*. [7] ont décrit cette atteinte sous forme d´anomalies touchant le complexe EP-chorio capillaire avec une infiltration inflammatoire des couches rétiniennes profondes et une infiltration œdémateuse maculaire possible voir une exsudation sous rétinienne. Des précipités sphériques sur l´interface vitréo-rétinienne correspondant à des agrégats cellulaires inflammatoires ont été également rapportés [5]. Dans notre cas, la *SS-OCT* en B scan a objectivé au niveau du foyer actif une désorganisation de la ligne IS/OS et des irrégularités de l´EP associé à une membrane de bruch intacte.

Par contre, au niveau du foyer cicatriciel, nous avons noté une désorganisation des couches rétiniennes externes et une rupture focale de la membrane de Bruch. Ceci pourrait correspondre au site initial de l´atteinte. En effet de nombreuses hypothèses ont conclu que la lésion initiale lors de la rétinochoroïdite toxoplasmique typique était une prolifération focale du parasite au niveau de la rétine responsable d´une rétinite nécrosante suivie d´une extension vers les différentes couches rétiniennes et depuis vers l´épithélium pigmentaire [8]. Ainsi, l´atteinte de toute l´épaisseur de la rétine objectivée par l´*OCT* caractérise la toxoplasmose oculaire et permet ainsi d´écarter d´autres diagnostics différentiels à savoir la choroïdite multifocale, choroïdite ponctuée interne ou la neurorétinite subaiguë unilatérale où la rétine externe est préservée [5]. Par rapport à l´*OCT* A, l´élargissement de la zone avasculaire centrale a été rapporté dans la littérature ainsi que la supériorité de cet examen dans la détection des néovaisseaux choroïdiens absents dans notre cas [9].

Différencier la RPE toxoplasmique des autres diagnostics est nécessaire afin de prescrire un traitement antiparasitaire adéquat, transformant le pronostic. Une récupération visuelle complète est la règle comme celle rapportée dans notre cas. Dans la littérature, la quasi-totalité des atteintes maculaires ont bien évolué sous traitement qui associait une bithérapie antiparasitaire et corticothérapie différée de 24 à 48h [1,5,6]. Notre patiente a été mise sous Azithromycine en monothérapie étant donné la rupture de pyriméthamine et de clindamycine en comprimés dans notre pays.

La récurrence de cette forme clinique et sa localisation maculaire préférentielle pourrait justifier une antibioprophylaxie secondaire. Parmi les conditions indiquant cette attitude nous citons le syndrome d´immunodéficience, un foyer menaçant la vision et une récurrence assez rapprochée dans le temps [10]. La rétinite ponctuée externe toxoplasmique n´a pas été considérée comme une indication, ceci peut être expliqué par la rare présentation clinique de cette forme souvent méconnue. Des études seraient nécessaires pour évaluer cette approche.

## Conclusion

La RPE toxoplasmique est une présentation rare de la toxoplasmose oculaire. L´imagerie multimodale permet de la différencier cette forme clinique des autres causes de rétinite unilatérale pour une prise en charge adéquate.
